# Identifying barriers to chronic disease reporting in Chicago Public Schools: a mixed-methods approach

**DOI:** 10.1186/1471-2458-14-1250

**Published:** 2014-12-06

**Authors:** Victoria Rivkina, David E Tapke, Lilliana D Cardenas, Blair Harvey-Gintoft, Stephanie A Whyte, Ruchi S Gupta

**Affiliations:** Office of Student Health and Wellness, Chicago Public Schools, 42 West Madison Street, Garden Level, Chicago, IL 60602 USA; Department of Pediatrics, Rush University Medical Center, 1653 W. Congress Parkway, Suite 770, Chicago, IL 60612 USA; Office of Tobacco and Chronic Disease Prevention, Maricopa County Department of Public Health, 4041 North Central Avenue, Suite 1400, Phoenix, AZ 85012 USA; Public Health Institute of Metropolitan Chicago (PHIMC), 180 North Michigan Avenue, Suite 1200, Chicago, IL 60601 USA; Center for Community Health, Feinberg School of Medicine, Northwestern University, 750 North Lake Shore Drive, Suite 670, Chicago, IL 60611 USA

**Keywords:** Asthma, Food allergy, Chronic disease, Schools nurses, Parents, Reporting, Barriers

## Abstract

**Background:**

Chronic disease among school-aged children is a public health concern, particularly for asthma and food allergy. In Chicago Public Schools (CPS), rates of asthma and food allergy among students are underreported. The aim of this study was to determine the barriers to chronic disease reporting as experienced by CPS parents and school nurses.

**Methods:**

A mixed-methods approach included focus groups and key informant interviews with parents and school nurses, and a cross-sectional survey was completed by parents. Qualitative data analysis was performed and survey data were analyzed to determine the significant demographic and knowledge variables associated with successfully completing the reporting process.

**Results:**

The three main barriers identified were 1) a lack of parental process knowledge; 2) limited communication from schools; and 3) insufficient availability of school nurses. Parents were significantly more likely to successfully complete the reporting process if they knew about special accommodations for chronic diseases, understood the need for physician verification, and/or knew the school nurse.

**Conclusions:**

These findings suggest that increasing parental knowledge of the reporting process will allow schools to better identify and manage their students’ chronic conditions. A parent-focused intervention informed by these results has been completed.

**Electronic supplementary material:**

The online version of this article (doi:10.1186/1471-2458-14-1250) contains supplementary material, which is available to authorized users.

## Background

Chronic disease among school-aged children is a major national public health concern. It is estimated that 15% to 20% of US children and adolescents are currently impacted by chronic disease [1], with asthma and food allergy among the most common. Both nationally and in Chicago, pediatric asthma prevalence is approximately 13% to 14% [2, 3], while pediatric food allergy prevalence is estimated at 8% to 10% [4, 5].

**Figure 1:**
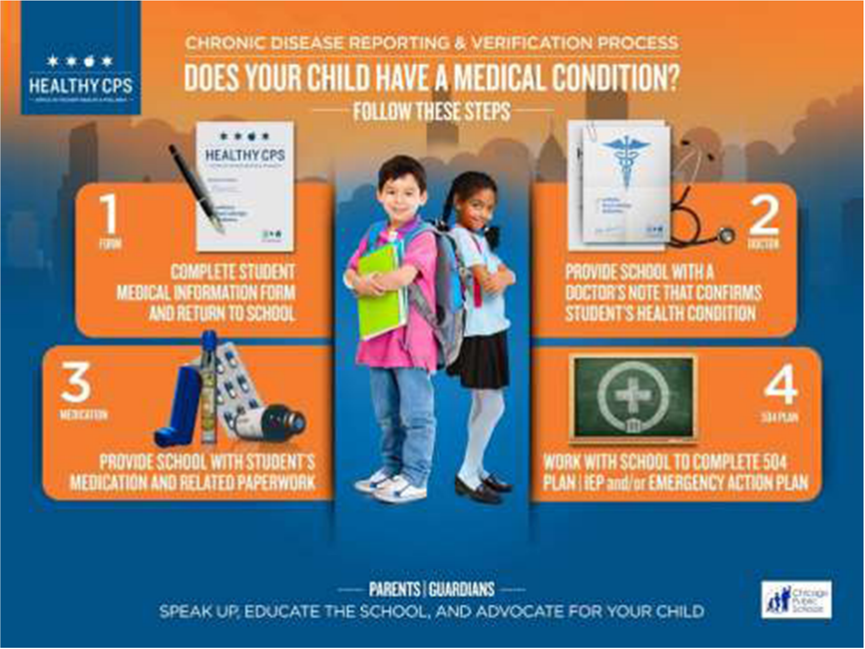
**CPS chronic disease reporting and verification process.**

In order for schools to meet the needs of students impacted by chronic disease, the students’ condition(s) must be reported by parents and verified by healthcare providers. In Chicago, the local school district requires parents to follow a four-step process for chronic disease reporting and verification (Figure 1). Only after a child’s condition is verified by a physician can it be entered into the district’s database and allotted clinical support services. Successful completion of the reporting and verification process is achieved through the development of an individualized action plan tailored to the child’s needs. Because both asthma and food allergy may cause potentially life-threatening reactions without warning, providing access to clinical services and ensuring the development of appropriate action plans is recognized as a critical task for all school districts [6–9].

Unfortunately, studies have shown that many chronic conditions remain underreported in schools [10, 11], and Chicago is no exception. As the third largest school district in the country, serving over 400,000 predominantly low-income and minority students [12], it is imperative for Chicago Public Schools (CPS) to maintain accurate records of its students’ chronic conditions. However, this study’s authors recently determined that physician-verified prevalence of asthma and food allergy is documented to affect only 4.5% and 1.0% of CPS students, respectively [13]. These rates, which fall far below both national and local rates of disease, demonstrate the gross underreporting of common chronic health conditions in CPS [2–5, 13]. Additionally, only 25% of students with asthma and 50% of students with food allergy have a school health management plan on file [13]. It is clear that the current documentation process is limited in its efficacy to identify students with chronic conditions in order to support their healthcare needs. Thus, the aim of this study was to identify barriers to chronic disease reporting and verification as experienced by CPS parents and school nurses.

## Methods

This study was conducted as part of the *Improving Chronic Disease Verification and Medication Access in Chicago Public Schools* research initiative, a partnership between Northwestern University and the CPS Office of Student Health and Wellness. It was approved by Northwestern University’s Institutional Review Board and the CPS Research and Review Board.

### Participants

Ten CPS partner schools were recruited to participate in the study based on geographic location, socioeconomic diversity of the student population, and administrative support. Because there were several components to the overall study, schools were able to select to what extent they would participate. The level of participation and demographics [12] for each school are presented in Table 1.

**Table 1:** **Research study participation and demographic information, by school**

School	Parent focus groups	Parent key informant interviews	School nurse key informant interviews	Parent surveys	% minority students	% low-income students*
**School A**	X		X	X	36.3%	17.5%
**School B**			X		99.8%	93.4%
**School C**		X	X		47.8%	10.1%
**School D**	X		X	X	94.7%	96.2%
**School E**	X		X	X	98.2%	98.5%
**School F**					94.4%	96.5%
**School G**	X	X	X	X	72.6%	52.8%
**School H**	X		X	X	36.9%	36.3%
**School I**		X	X	X	79.0%	51.0%
**School J**					98.4%	93.0%
**District**	N/A	N/A	N/A	N/A	90.7%	85.0%

*Students who qualify for the free/reduced lunch program.

Through posted recruitment flyers and emails from participating school principals, parents from each school were invited to participate in focus groups and key informant interviews. Parents to whom the study was applicable and interesting then attended scheduled focus groups or set up meetings to participate in key informant interviews with research personnel.

School nurses were invited to participate in key informant interviews through email recruitment by research personnel. Nurses interested in study participation set up in-person meetings with researchers in order to complete the interview.

Parents who completed the survey questionnaire did so during report card pick-up day at six of the ten schools at which principals had agreed to have researchers stationed. All parents who encountered research personnel were asked to complete the survey. This recruitment method utilized a convenience sample of parents.

### Procedure

Five parent focus groups were conducted between November 2012 and March 2013. Each focus group had between three and eight participants and consisted primarily of parents of children with asthma and/or food allergy. The focus groups lasted between 33 and 84 minutes, were conducted in English or Spanish as needed, and were recorded and transcribed. All participants completed a consent form and received a $20 Target gift card as an incentive for participation. Focus group questions assessed knowledge of the chronic disease reporting and verification process, personal experiences with reporting and managing children’s conditions at school, and feedback on a draft of the parent survey (Table 2).

**Table 2:** **Sample focus group and key informant interview questions**

Domain	Focus groups	Parent interviews	Nurse interviews
**Food allergy reporting**	Let’s go around and share personal experiences with chronic disease verification at CPS.	Please share your experience with chronic disease reporting and verification at your child’s school?	What process does your school use to identify students with chronic conditions?
	Have you all seen this Student Medical Information form?	Did you receive this Student Medical Information form from the school?	How does your school use the Student Medical Information form?
	Do you have any suggestions for improving the form?	Did you fill this form out and return it to the school?	How can this process be improved?
**Physician verification**	Did everyone provide their school with a signed physician’s diagnosis of their child’s chronic disease?	Did you get physician verification of your child’s chronic condition?	How do you go about getting physician verification from parents/caregivers?
	Has anyone encountered barriers when trying to verify your child’s condition?	Did you face any problems when trying to provide this information to the school?	What are your barriers to getting chronic disease verification?
**Access to medication**	Who has supplied their child’s school with chronic disease medication?	Does your child have his/her chronic disease medication at school?	Can you share your experiences related to medication access?
	What are some ways that barriers can be removed so all children have access to their medication at school?	Did you encounter any barriers at school that made it difficult to provide your child’s medication?	Do all students at your school have the prescribed medication the need to manage their condition?
	Do your children carry their medication on them?	Does your child always have his/her medication with him/her?	Do you have any suggestions for improving this process?
**Action plan development**	Has anyone obtained a 504 Plan for their child?	Did you set up a 504 Plan for your child?	What is your experience with setting up 504 Plans for your food allergic students?
	Did you encounter any barriers during this process? How can those barriers be reduced?	What were some of the barriers you faced when trying to set up an action plan?	What works well and what is challenging about this system?
**Communication**	What are the ways in which your children’s school sends home health-related information?	How do you typically receive health-related information from your child’s school?	What is communication like between you and the students’ parents/caregivers?
	How would you like to receive information from the school?	Is this the most convenient method for you? What would be most preferable?	How can communication be improved?
**Survey development**	Do these questions capture the information we’re looking to collect?	Are these questions relevant to you as a parent?	N/A
	Is anything on this survey confusing?	Are we missing anything? Should anything be removed	N/A

Five key informant interviews were conducted with parents between December 2012 and September 2013. The interviews lasted between 29 and 57 minutes, were conducted in English, and were recorded and transcribed. All participants completed a consent form and received a $25 Target gift card as an incentive for participation. Parent key informant interview questions assessed knowledge of the chronic disease reporting and verification process, personal experiences with reporting and managing children’s conditions at school, and feedback on a draft of the parent survey (Table 2).

Seven key informant interviews were conducted with school nurses between May and September 2013. The interviews lasted between 14 and 37 minutes, were conducted in English, and were recorded and transcribed. All participants completed a consent form and received a $25 Target gift card as an incentive for participation. School nurse key informant interview questions assessed personal experiences with verifying and managing children’s conditions at school as well as perceived barriers to compliance (Table 2).

### Instruments

Parent feedback from focus groups and key informant interviews was used to finalize a survey instrument designed to gain a broader perspective of barriers to chronic disease reporting and verification as experienced by CPS parents. Surveys distributed to all parents contained items assessing perceptions of school-to-parent communication, as well as basic demographic data. For parents of children with chronic diseases, additional questions assessed parental knowledge of the chronic disease reporting process as well as attitudes toward school health services. Surveys were offered in both English and Spanish. Parents provided consent by reading information about the study and checking a box on the front cover of the questionnaire. Please see Additional file 1 for the English language version of the survey questionnaire. No financial incentive was provided for completing the survey.

### Data analysis

All focus group and key informant interview audiotapes were transcribed by volunteer college students. A coding scheme based on eight content domains was developed after reviewing initial transcripts. Content domains were as follows: 1) child, 2) communication, 3) condition, 4) education, 5) knowledge, 6) nurse, 7) parental attitude, and 8) process. Once an initial round of coding was completed, codes were modified and expanded to more accurately capture emerging themes. Two reviewers independently coded each transcript, coding discrepancies were reconciled, and master documents were created after reconciliation. All coding and subsequent qualitative data analysis and extraction was done with version 7.1.6 of the *ATLAS.ti* qualitative data analysis software (ATLAS.ti, 2013, Berlin, Germany).

Survey data from parents of children with chronic conditions were stratified according to whether or not each parent had successfully completed the reporting process. Successful completion was defined as having an action plan on file with the school, such as a 504 plan or an Individualized Education Program (IEP) with health accommodations. Chi square tests of association were used to detect significant differences between parents who had or had not successfully completed the process with regard to demographic characteristics and process knowledge variables. A logistic regression model was then estimated based on the demographic or knowledge variables found to be significantly associated with completing versus not completing the process. A random effects logistic regression model to test for potential correlation within schools was also estimated, but the results did not change. Statistical analyses were completed with version 22.0 of the SPSS statistical software (IMB SPSS, 2013, Armonk, NY).

## Results

### Parent focus groups & interviews

Based on the parent focus groups and key informant interviews conducted, barriers to chronic disease reporting and verification were predominantly comprised of three overarching themes: 1) a lack of parental process knowledge; 2) limited communication from schools; and 3) insufficient availability of school nurses. Examples of specific responses, arranged by theme, are presented in Table 3.

**Table 3:** **Selected parent and school nurse quotes**

Theme	Quotation
**Parents**	
**Lack of parental knowledge**	"Maybe there could be workshops for parents, or places we could go and find out more information. Whether it’s something to have at the beginning of the year or [have] over the summer for us to go and find out what works best. Because if you don’t know that your child needs something else, you’re going to just scrape by with what you have."
	"If you’re a parent who suspects that your kid has asthma, allergy or a chronic illness, what do you do? Some parents don’t know."
	"You know, unfortunately, we are handed these papers [but] no one is really consulting us as to what they mean."
**Unsatisfactory communication from schools**	"I guess the overall experience will go back to communication from the school in the beginning. It needs to be really clear: here is what you need to know before August…what your rights are. This is what you really need to think about!"
	"We don’t know who to go to if we do have any issues. I have been fortunate enough where my children have been healthy and doing fine, but I can’t take that for granted at all."
	"I don’t know enough. I feel like we’ve come so far from where we used to be…but yet, there’s still so much that’s not being communicated to parents. We should know exactly what’s going on in the cafeteria…we should know who’s watching [our children]."
	"It seems like it needs to be clear as day on the website. For me, as a new parent, it wasn’t clear where to click on the website to get [health forms]. So I still don’t know if I filled out the correct forms or not."
**Limited school nurse availability**	"Maybe [individual schools] can have something that says, ‘This is your school nurse.’ I know a lot of people don’t even know who the school nurse is. Like me…I didn’t know who the school nurse was here and what day she’s available. Then, if you have any health concerns, you know who to direct those to."
	"There’s no question that there needs to be a nurse here. I don’t understand why there [are] part time nurses and always less than needed. In a system like this, it doesn’t make sense to me."
**Success of parent advocates**	"As a parent going through this system, you don’t wait for anything. If you’re not the advocate, nothing will happen."
	"It would also be important for parents to know their rights about what can be offered to them and to their children. Coming in, [I] was aware and was ready…but what about the other parents? There’s lots of them, I’m sure."
	"One thing I would like to mention is I was able to have my papers ready by the first day of school. That was because I did my own research through different parent forums through the city and CPS parent forums. I read information and then I specifically had asked for forms during registration."
**School nurses**	
**Lack of parental knowledge**	"One thing that I’ve often wondered is how aware the parents are. I think the parents need to be more educated on this stuff because they really are not informed. They have no idea that this kind of thing is happening."
	"Sometimes [parents] do not understand the 504 process, the value of it, or the addition of the medical side of the IEP. When they have an IEP it’s very easy because they have an annual IEP meeting and I will be present. With the 504, they don’t understand why."
**Limited nursing resources and time constraints**	"I don’t have the time to really do the job I would like to do, and that is actually the case in my other two schools as well. We don’t have enough school hours; we have cut back [by] almost half. We have twice as many responsibilities within CPS."
	"I think more nursing hours would help a ton with dealing with 504 s. And I think [what] I mentioned before is [the value of] having a full time person just updating the telephone numbers, the email of parents, etc., so we have current ways to contact parents."
	"I can’t even finalize the meetings or the notes. I simply do not have the time to figure all of that out. So if I ask [a parent] for a meeting and she doesn’t set it up right away, it can be hard to remember."

#### Lack of parental process knowledge

Many parents struggled to identify the essential steps and corresponding paperwork necessary for chronic disease reporting and verification in CPS. For example, participants did not routinely know to complete the annual Student Medical Information form, to obtain physician verification, to supply the school with their child’s medication, or to work with the school to complete the appropriate action plan for their child. Additionally, parents did not know whom to contact at their child’s school with questions, nor were they aware of the timeline for completing the reporting and verification process. Parents were eager to better understand the process, and frequently requested more education from the schools as well as easier access to necessary forms.

#### Limited communication from schools

Parents often felt that schools did not clearly communicate the steps that needed to be taken in order to formally report and verify a child’s chronic health condition. Without this basic understanding, parents expressed feeling lost and helpless. Suggestions for improving school-to-parent communication included: 1) providing checklists to clearly delineate the steps and associated forms of the chronic disease reporting and verification process; 2) offering a more concise timeline of when each step in the process should be completed; and 3) holding parent workshops or information sessions to update parents on the process and allow for questions/feedback. Proposed school-to-parent communication methods included email, telephone, having information sent home with students, and posting material on the school website.

#### Insufficient availability of school nurses

Parents were universally concerned about the lack of availability of school nurses in CPS, all of whom spend only one or two days per week at each assigned school. Parents found this to be problematic for disease management in general, and particularly concerning for the management of emergency situations such as severe asthma exacerbations or food-induced anaphylaxis. The absence of trained medical personnel on the premises at all times was a source of anxiety and frustration for parents given the unpredictable and potentially life-threatening nature of these events. Furthermore, the lack of full-time nursing staff at school also created logistical challenges for parents, many of whom reported difficulty setting up or completing 504/IEP meetings. Many parents stated that they had neither met their child’s school nurse nor had reliable contact information for him or her.

### Nurse interviews

Based on the seven interviews conducted, participating school nurses had worked in the district between nine and 22 years and covered three to five schools each. Nurses acknowledged many of the same challenges expressed by parents and identified barriers that fell into two predominant themes: 1) a lack of parental process knowledge and 2) insufficient resources and time constraints. Examples of specific responses, arranged by theme, are presented in Table 3.

#### Lack of parental process knowledge

CPS school nurses similarly acknowledged that many parents do not have a basic understanding of the chronic disease reporting and verification process. Furthermore, they felt that parents often did not appreciate the importance of completing the process in relation to their child’s health at school. This was often attributed to limited parent outreach and education on the part of the school. Parents with less process knowledge were described as being much less likely to properly complete health-related forms, provide physician verification of their child’s chronic condition, or attend meetings to complete necessary action plans for their child.

#### Insufficient resources and time constraints

All nurses interviewed were assigned to multiple CPS schools and described the challenges of managing the chronic disease reporting process for a large volume of students. They found that there was simply not enough time to adequately educate parents about the process or to follow-up with parents who had not completed all necessary steps. Additional barriers to successful chronic disease management included a time-consuming data entry process and a lack of current parent contact information.

### Parent surveys

A total of 283 parent surveys were collected from seven partner schools, including 72 from parents of children with chronic diseases (25.4%). This is representative of the 25% of the overall CPS student population that is estimated to have a chronic condition [12, 13]. Among parents of children with chronic disease, respondents were most often female (91.7%), under 45 years old (79.2%), self-reported as White/Other (47.2%), and had graduate level education (42.1%). In addition, 52.8% of these parents reported having an action plan for their child on file with the school (Table 4).

**Table 4:** **Demographic variability of parent survey respondents**

Variable	Frequency, n (%)		
	All parents	Parents who completed process	Parents who did not complete process
	N = 72	N = 38	N = 34
**Age**			
≤ 44	57 (79.2%)	30 (78.9%)	27 (79.4%)
≥ 45	15 (20.8%)	8 (21.1%)	7 (20.6%)
**Gender**			
Male	6 (8.3%)	3 (7.9%)	3 (8.8%)
Female	66 (91.7%)	35 (92.1%)	31 (91.2%)
**Race/Ethnicity**			
African American	13 (18.1%)	7 (18.4%)	6 (17.6%)
Hispanic/Latino	25 (34.7%)	11 (28.9%)	14 (41.2%)
White/Other	34 (47.2%)	20 (52.6%)	14 (41.2%)
Education level*			
High school or less	23 (31.9%)	14 (36.8%)	9 (26.5%)
Some college	18 (25.0%)	9 (23.7%)	9 (26.5%)
Graduate	31 (43.1%)	15 (39.5%)	16 (47.1%)
Household income**			
< $50,000	38 (52.8%)	20 (52.6%)	18 (52.9%)
> $50,000	34 (47.2%)	18 (47.4%)	16 (47.1%)
**Child insurance**			
Private	32 (44.4%)	15 (39.5%)	17 (50.0%)
Public or none	40 (55.6%)	23 (60.5%)	17 (50.0%)

*Parents with missing data (n = 4) were assumed to have high school education or less.

**Parents with missing data (n = 5) were assumed to be lower income (<$50,000).

#### Process knowledge

Only half (50.0%) of the parents of children with chronic conditions were aware that special accommodations were available for their children or were familiar with 504 and/or IEP plans. Many were also unaware of the need for physician verification of the child’s condition (26.4%), and a large portion did not know their child’s school nurse (41.7%) (Table 5).

**Table 5:** **School health knowledge variability of parent survey respondents**

Variable	Frequency, n (%)		
	All parents	Parents who completed process	Parents who did not complete process
	N = 72	N = 38	N = 34
Know about special accommodations**			
Yes	36 (50.0%)	27 (71.1%)	9 (26.5%)
No	36 (50.0%)	11 (28.9%)	25 (73.5%)
Know about 504/IEP plans**			
Yes	36 (50.0%)	28 (73.7%)	8 (23.5%)
No	36 (50.0%)	10 (26.3%)	26 (76.5%)
Know about physician verification**			
Yes	53 (73.6%)	37 (97.4%)	16 (47.1%)
No	19 (26.4%)	1 (2.6%)	18 (52.9%)
know who school nurse is**			
Yes	42 (58.3%)	33 (86.8%)	9 (26.5%)
No	30 (41.7%)	5 (13.2%)	25 (73.5%)

**Indicates significance of P < .0001.

No significant associations were observed between successful completion of the reporting/verification process and respondent demographics, including parent age, gender, race/ethnicity, education, household income, or child’s insurance status. However, parents were significantly more likely to complete the process if they were aware of special accommodations for chronic diseases, were familiar with 504/IEP plans, understood the need for physician verification, and/or knew the school nurse. Both understanding the need for physician verification and knowing the school nurse remained significantly predictive of successful process completion after adjusting for potential confounders (OR 13.45, 95% CI 1.25 – 144.85 and OR 9.70, 95% CI 2.36-39.75, respectively) (Table 6).

**Table 6:** **Knowledge variables predictive of successful process completion**

Variable	Odds ratio [95% CI]*	P value
Know about special accommodations	1.71 [0.38 – 7.70]	0.485
Know about 504/IEP plans	3.59 [0.85 – 15.11]	0.082
Know about physician verification	13.45 [1.25 – 144.85]	0.032
Know who school nurse is	9.70 [2.36 – 39.75]	0.002

* 95% CI = 95.0% Confidence Interval.

#### Attitudes and beliefs

Parents whose children had chronic conditions more frequently rated school-to-parent health-related communication poorly compared to parents of children without chronic conditions (33.3% versus 12.8%, respectively). Regardless of child’s chronic disease status, communication preferences included email communication (30.0%), written information sent home with the child (25.8%), electronic information via the CPS parent portal (10.9%), and newsletters (11.5%). The majority of parents had internet access at home (88.0%) and an active email address (89.4%).

Parents of children with chronic conditions also more frequently perceived their child’s health to be a lower priority for the school compared to parents of children without chronic conditions (20.9% versus 10.9%, respectively). Regardless of their child’s disease status, most parents agreed that having a full-time school nurse was very important (68.6%) and that this nurse should be in the school five days per week (78.1%).

## Discussion

To our knowledge, this is the first study to utilize a mixed-methods approach to examine barriers to chronic disease reporting and verification among parents and school nurses in a large, urban school district. It was determined that the three most pervasive barriers to successful chronic disease reporting and verification in CPS are: 1) a lack of parental process knowledge; 2) limited communication from schools; and 3) insufficient school nurse availability. These barriers likely contributed to the fact that nearly half of the parents of children with chronic conditions who participated in this study did not have an appropriate action plan on file for their child. Notably, successful completion of the chronic disease reporting and verification process was significantly associated with parental knowledge variables but not with respondent demographics. Specifically, understanding the need for physician verification and knowing the school nurse were most predictive of successful process completion.

It is imperative to improve school-based chronic disease reporting systems. With regard to asthma and food allergy, reliable disease reporting is critical to ensure proper care in the event of a medical emergency. Increased reporting and verification can lead to improved health outcomes by reducing exposure to triggers, promoting early recognition of adverse reactions, and facilitating prompt administration of appropriate medications. Unfortunately, data indicate that only 25-28% of students with asthma have written management plans on file at school [14, 15], and access to quick relief medication may be as low as 14% [16]. Timely administration of epinephrine auto-injectors has been shown to improve morbidity and mortality in the event of food-induced anaphylaxis [17–19]. However, it has been estimated that only 25-28% of children with food allergy have individually prescribed access to an epinephrine auto-injector while at school [20–22].

While it is well established that urban populations and racial minorities disproportionately suffer from asthma and associated morbidities [21, 23, 24], this study did not find significant associations between demographic variables and the likelihood of establishing an action plan at school. There are conflicting results in the literature regarding the impact of these factors on the management of asthma in schools. An Alabama school study found no association between demographic factors and medication access [16], while a survey of school nurses in New York state demonstrated a statistically significant decrease in access to quick-relief asthma medication at lower socioeconomic levels [10]. It is conceivable that parents of a lower socioeconomic background may face financial barriers to the completion of the disease reporting/verification process, such as limited access to physicians to complete verification paperwork or difficulty obtaining necessary medications for use while at school. However, this study found no such associations.

A lack of parental process knowledge, limited school-to-parent communication, and insufficient nursing resources were identified as barriers to the reporting, and therefore proper management, of chronic conditions in schools. These barriers are consistent with those previously described in the literature. Major et al. conducted focus groups with elementary school nurses and found four major barriers to asthma management: 1) lack of education for nurses, parents, and physicians; 2) lack of communication; 3) lack of nursing resources; and 4) lack of respect [25]. In other studies, school staff identified a lack of consistent communication strategies [26] as well as a lack of nursing time and funding as significant obstacles to asthma management [14, 15]. Additional barriers reported by school nurses included inadequate contact information for parents and difficulty having parents properly complete health forms [14, 15, 26, 27].

Because the results of this study indicated that parental education has the potential to improve school-based identification of children with chronic conditions, an intervention focusing on increasing parental process knowledge has been carried out. Influenced by the Chronic Care Model [28–32], this intervention was developed to create informed, activated parents who are able to complete the district’s four-step chronic disease reporting and verification process and empowered to advocate for their children’s medical needs at school. This evidence-based, multi-tiered intervention was comprised of three parts: 1) in-person parent education at the Local School Council meetings of this study’s partner schools; 2) the distribution of educational print material; and 3) an online toolkit for parents housed on the Office of Student Health and Wellness website. Additionally, each of the district’s 675 schools received a 2’ × 3’ laminated poster of Figure 1 to be displayed in locations frequented by parents. By directly targeting the barriers identified by CPS parents and school nurses, this intervention aimed to improve chronic disease reporting and verification by empowering parents to advocate for their child’s medical needs at school.

Although increasing nursing resources to provide better support services for students with chronic conditions would be beneficial, funding constraints often limit a school district’s ability to hire more staff. In regard to CPS health-related policy, the district has already implemented comprehensive Asthma Management and Food Allergy Management policies, as well as an Administration of Medication policy that includes guidelines on using district-issued undesignated epinephrine auto-injectors for any person experiencing an anaphylactic emergency on school grounds [33–37]. As there is currently no comparable policy to stock district-issued quick-relief asthma medication [37, 38], this is an area where the district can continue to improve in the near future.

This study is not without its limitations. While focus group and interview data offer insight into members of a particular community, it is important to note that perspectives may be influenced by the participant’s geographic region and unique experience with specific CPS schools. Consequently, findings and conclusions may not be generalizable to other school districts state- or nation-wide. Additionally, White, highly educated parents and privately insured children were overrepresented in this study as compared to the general CPS population. Although one could hypothesize that a more educated group of respondents would be better able to navigate the district’s reporting and verification process to establish an action plan, the fact that this was not the case in this study underscores the need for increased parental education across racial/ethnic, financial, and educational lines. Other limitations include the fact that survey data analysis was restricted to a small sample size of parents of children with chronic diseases, and that there was a need to impute missing data. This limited the ability to perform statistical analysis on sub-groups and examine action plan status by disease state.

## Conclusions

To our knowledge, this was the first study to utilize a mixed-methods approach to determine the barriers to chronic disease reporting and verification perceived by parents and school nurses in a large, urban school district. The three most pervasive barriers identified were 1) a lack of parental process knowledge; 2) limited communication from schools; and 3) insufficient availability of school nurses. In addition, this study found that knowledge variables, rather than demographic characteristics, were significantly associated with successful process completion. Findings suggest that improving parental education will lead to increased chronic disease reporting in the district, thereby improving disease management at school. Efforts to this end are currently under way.

## Electronic supplementary material

Additional file 1: **Parent survey on asthma, food allergy, and diabetes management in Chicago Public Schools.** (PDF 141 KB)

## Abbreviations

CPS: Chicago Public Schools.
