# Supplementary material for: Identifying barriers to chronic disease reporting in Chicago Public Schools: a mixed-methods approach
Source: BMC Public Health. 2014 Dec 6;14:1250. doi: 10.1186/1471-2458-14-1250 (PMC4265490; doi:10.1186/1471-2458-14-1250)
Supplement: Supplementary file 1 — Additional file 1: Parent survey on asthma, food allergy, and diabetes management in Chicago Public Schools. (PDF 141 KB) [file 12889_2014_7343_MOESM1_ESM.pdf]

Parent Survey on Asthma, Food Allergy, and Diabetes Management in Chicago Public Schools

This survey is part of a research study conducted by the
Chicago Public Schools Office of Student Health and Wellness and
Northwestern University Feinberg School of Medicine.
(Principal Investigator: Ruchi Gupta, MD, MPH; IRB STU00062953)

Participation in this research study involves completion of this anonymous survey (10 minutes). Participation is voluntary; you may skip any questions or withdraw at any time by simply not submitting the survey.

If you have any questions about the research, you can contact:
Dr. Ruchi Gupta at (312) 503-3005 or r-gupta@northwestern.edu
Lilliana DeSantiago at (773) 553-5135 or ladesantiago@cps.edu

Asthma, food allergy, and diabetes are underreported to CPS schools. It is important that schools know which students have these conditions so the child has support, access to services, and medication while at school.

The goal of this survey is to get your experience reporting your child's asthma, food allergy, and/or diabetes to the school. We also want to hear your ideas for how this reporting process can be improved. The results of this survey will be used to make recommendations to your school and the district to improve the way asthma, food allergy, and diabetes are managed.

☐ **By checking this box I consent to participate in this research study.**

1. Do you have any children who attend Chicago Public Schools that have asthma, food allergy or diabetes?
    - ☐ Yes
    - ☐ No → Thank you for your interest. Please see a member of our research team to get the appropriate survey.

Please tell us about your children with asthma, food allergy, and/or diabetes that currently attend CPS grades Pre-K – 12th grade.

	**Child # 1**	**Child # 2**	**Child # 3**
**2. What grade is your child currently in?**	_____ grade	_____ grade	_____ grade
**3. What school does your child attend?**			
**4. Has he/she ever experienced a severe allergic reaction?**	☐ Yes<br>☐ No	☐ Yes<br>☐ No	☐ Yes<br>☐ No
**5. To which foods is he/she CURRENTLY allergic?**	☐ Milk<br>☐ Egg<br>☐ Peanut<br>☐ Soy<br>☐ Wheat<br>☐ Sesame seeds<br>☐ Tree nut (almonds, pecans, cashews, etc.)<br>☐ Fin fish (salmon, tuna, etc.)<br>☐ Shellfish (shrimp, crab, lobster, etc.)<br>☐ Other	☐ Milk<br>☐ Egg<br>☐ Peanut<br>☐ Soy<br>☐ Wheat<br>☐ Sesame seeds<br>☐ Tree nut (almonds, pecans, cashews, etc.)<br>☐ Fin fish (salmon, tuna, etc.)<br>☐ Shellfish (shrimp, crab, lobster, etc.)<br>☐ Other	☐ Milk<br>☐ Egg<br>☐ Peanut<br>☐ Soy<br>☐ Wheat<br>☐ Sesame seeds<br>☐ Tree nut (almonds, pecans, cashews, etc.)<br>☐ Fin fish (salmon, tuna, etc.)<br>☐ Shellfish (shrimp, crab, lobster, etc.)<br>☐ Other
**6. How would you rate your child's asthma?**	☐ Mild<br>☐ Moderate<br>☐ Severe	☐ Mild<br>☐ Moderate<br>☐ Severe	☐ Mild<br>☐ Moderate<br>☐ Severe
**7. What type of diabetes does your child have?**	☐ Type 1<br>☐ Type 2<br>☐ Don't know	☐ Type 1<br>☐ Type 2<br>☐ Don't know	☐ Type 1<br>☐ Type 2<br>☐ Don't know

8. Why were you unable to report your child's health condition to their school this year?

____

____

____

Please think of CHILD #1 (from the chart above) when answering questions 9-45.

The Student Medical Information Form was created to better identify students with health conditions so that they can access services and medications at school. This form should be sent to every student at the start of the school year on an annual basis by your school.

9. Did your school send you the CPS Student Medical Information form this school year? (see picture below)
   - ☐ Yes
   - ☐ No

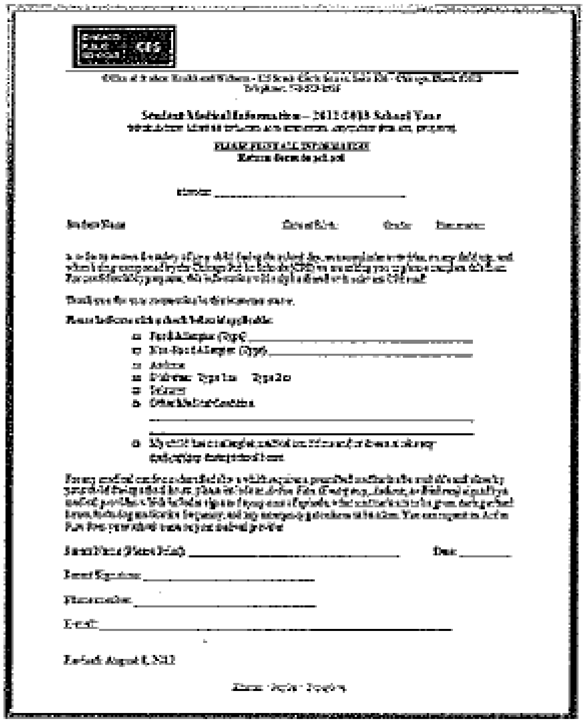

10. Did you return your completed Student Medical Information form back to the school?
    - ☐ Yes
    - ☐ No → skip to question #12

11. After turning in the Student Medical Information form, were you contacted by the school nurse about the condition(s) you marked on the form?
    - ☐ Yes, my child was sent home with additional forms to get filled out
    - ☐ Yes, I was contacted by phone
    - ☐ No, I was not contacted by the school

12. What was the reason you were unable to return the form to the school?
    - ☐ My child does not take medication at school
    - ☐ I already reported my child's health condition to the school; the school already knows
    - ☐ I do not think the school should ask about my child's health condition
    - ☐ I did not know where to return the form
    - ☐ I forgot
    - ☐ Other: ______________________________

13. Are you aware that if your child has asthma, food allergy, or diabetes, they may be eligible to get special accommodations during the school day (such as extra time to walk between classes, water breaks, food substitutions, etc.)?
    - ☐ Yes
    - ☐ No

14. Can you suggest any changes to make it easier for parents to complete the Student Medical Information form? Please select ALL that apply.
    - ☐ Ask parents to fill out a <u>paper</u> form at school events like open house or report card pick-up
    - ☐ Ask parents to fill it out on a <u>computer</u> at school
    - ☐ Ask parents to fill out the form through <u>email</u>
    - ☐ Send it home with other required forms at the beginning of the school year
    - ☐ Other: ______________________________
    - ☐ I don't know

Medication Access

**15. Circle the medications your child uses for their asthma, food allergy, or diabetes:**	Epinephrine auto-injector:<br><br>EpiPen<br>Auvi-Q<br>Twinject	Antihistamine:<br><br>Benadryl<br>Claritin<br>Zyrtec<br>Allegra<br>Other	Asthma medication:<br><br>Inhaler<br>Puffer<br>Pump	Insulin pump	Other:
**16. Circle the medications your child self-carries at school:**	Epinephrine auto-injector:<br><br>EpiPen<br>Auvi-Q<br>Twinject	Antihistamine:<br><br>Benadryl<br>Claritin<br>Zyrtec<br>Allegra<br>Other	Asthma medication:<br><br>Inhaler<br>Puffer<br>Pump	Insulin pump	Other:
**17. Circle the medications the school holds (in main office, nurse's office, classroom, etc.):**	Epinephrine auto-injector:<br><br>EpiPen<br>Auvi-Q<br>Twinject	Antihistamine:<br><br>Benadryl<br>Claritin<br>Zyrtec<br>Allegra<br>Other	Asthma medication:<br><br>Inhaler<br>Puffer<br>Pump	Insulin pump	Other:

18. If your child does not have their medication at school, please let us know why. Please select ALL that apply.
    - ☐ My child doesn't take medication
    - ☐ My child rarely needs his/her medication
    - ☐ It is too expensive to supply medication at school
    - ☐ I can't get a prescription for extra medication to keep at school
    - ☐ Other: ______________________________

19. What would make it easier for your child to have access to medication at school? Please select ALL that apply.
    - ☐ Medication consent forms online
    - ☐ Extend the expiration date on the forms so annual renewal isn't needed
    - ☐ School would allow my child to carry and self-administer
    - ☐ School would have a secure place to store medication
    - ☐ Other: ______________________________

Medication Access

20. Does your child have an action plan at school? See below for action plan options.
 ☐ Yes
 ☐ No

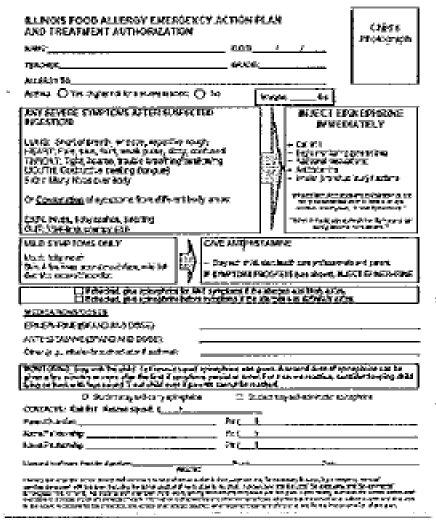
ILLINOIS FOOD ALLERGY EMERGENCY ACTION PLAN AND TREATMENT AUTHORIZATION

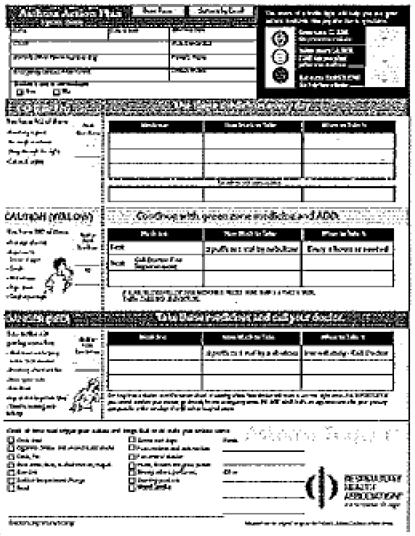
Asthma Action Plan

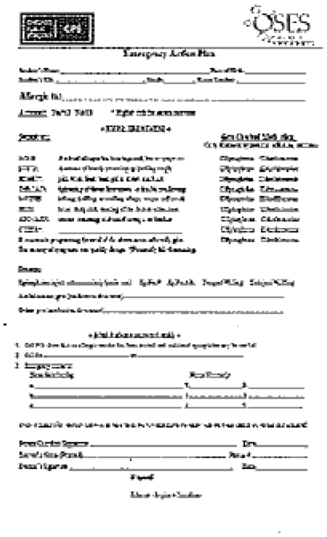
Emergency Action Plan

Physician Verification

21. Are you aware that CPS requires a physician (health care provider) to confirm that your child has asthma, food, allergy, or diabetes for your child's condition to be documented and for him/her to qualify for accommodations and a 504 Plan?
 ☐ Yes
 ☐ No

22. Did you provide the school with physician confirmation of your child's asthma, food food allergy, and/or diabetes?
 ☐ Yes
 ☐ No

23. If not, why? Please select ALL that apply.
 ☐ I did not know if was required
 ☐ My child does not have a regular physician
 ☐ It takes too long to get an appointment with the physician
 ☐ I do not want to give the school this information
 ☐ Other: ______________________________

504/IEP Plan

24. Do you know what a Section 504 Plan is?
    - ☐ Yes
    - ☐ No

A Section 504 Plan is a legal document filled out by the parent/caregiver, school nurse, and case manager. The plan outlines accommodations that the child needs during their school day for their asthma, food allergy, or diabetes. It can include things as simple as "Student needs extra time walking between classes so they don't trigger an asthma attack," "Student can carry water bottle to make sure they drink extra water at school," "Student can carry and self-administer medication," or "Student must sit at the peanut- free table at lunch." Contact your school nurse if you have any questions.

25. Does your child have a Section 504 Plan or Individualized Education Plan (IEP) on file?
    - ☐ Yes, my child has a 504 Plan → skip to question #29
    - ☐ Yes, my child has an Individualized Education Plan (IEP)
    - ☐ No → skip to question #28

26. Does your child's IEP include accommodations for his/her asthma, food allergy, or diabetes?
    - ☐ Yes
    - ☐ No

27. Are you interested in learning more about your child's options for a 504 Plan?
    - ☐ Yes
    - ☐ No

28. How would you like to receive information on Section 504 Plans or Individualized Education Plans?
    - ☐ Parent workshops
    - ☐ Parent newsletters
    - ☐ From my school nurse or case manager
    - ☐ From the principal
    - ☐ My school's website
    - ☐ The CPS website

29. Can you suggest any other changes to make it easier for parents to report their child's condition, get medication, an action plan, and a 504 Plan at school?

____

____

Communication

30. Do you know who your child's school nurse is?
    - ☐ Yes
    - ☐ No

31. Do you feel confident that the staff at your child's school is knowledgeable on the management of your child's health condition?
    - ☐ Yes
    - ☐ No
    - ☐ Indifferent

32. Who is responsible for managing your child's condition during the school day?
    - ☐ School nurse
    - ☐ Classroom teacher
    - ☐ Delegated Care Aide
    - ☐ Case manager/counselor
    - ☐ Main office clerk
    - ☐ My child self-manages
    - ☐ Other: ______________________________

33. What resources would help you better document and manage your child's condition at school? Please select ALL that apply
    - ☐ Better communication with school nurse
    - ☐ Knowing my child's options for care and accommodation during the school day
    - ☐ School staff who speak my primary language
    - ☐ Knowing the school policies for asthma, food allergy, and/or diabetes management
    - ☐ Less paperwork
    - ☐ Online communication system
    - ☐ Other: ______________________________

34. How would you rate your school's general communication with parents?
    - ☐ Very poor
    - ☐ Poor
    - ☐ Fair
    - ☐ Good
    - ☐ Very good

35. How would you rate your school's communication about <u>health</u> issues?
    - ☐ Very poor
    - ☐ Poor
    - ☐ Fair
    - ☐ Good
    - ☐ Very good

36. To what extent do you feel your child's school values health and wellness?
- ☐ Very poor
- ☐ Poor
- ☐ Fair
- ☐ Good
- ☐ Very good

37. Have you ever had contact with your child's school nurse?
- ☐ Yes
- ☐ No

38. How important is it for your school to have a full time nurse?
- ☐ Not important
- ☐ Somewhat important
- ☐ Important
- ☐ Very important

39. How often do you think the school nurse should be at school?
- ☐ 1 day per week
- ☐ 2 days per week
- ☐ 3 days per week
- ☐ 4 days per week
- ☐ 5 days per week

40. Has your child ever had any of the following health issues at school?
- ☐ Fever
- ☐ Vomiting
- ☐ Injury
- ☐ Allergic reaction
- ☐ Rash
- ☐ Asthma attack
- ☐ Other: ________________________________

41. Does your child have any other health conditions? Please specify below.

____

42. What type of health insurance does your <u>child</u> have?
- ☐ Medicaid
- ☐ KidCare
- ☐ Private (HMO, PPO, etc.)
- ☐ Other: ________________________________
- ☐ None

Please tell us about yourself.

43. What is your gender?
    - ☐ Male
    - ☐ Female

44. How old are you?
    - ☐ 18-24 years old
    - ☐ 25-44 years old
    - ☐ 45-64 years old
    - ☐ 65+ years old

45. What is your race/ethnicity? Please mark ALL that apply.
    - ☐ White
    - ☐ African American
    - ☐ Hispanic/Latino
    - ☐ Asian
    - ☐ Other: ______________________________

46. What is the highest level of education you have completed?
    - ☐ Less than high school
    - ☐ High school
    - ☐ 2 year college
    - ☐ 4 year college
    - ☐ Graduate degree

47. Which of the following categories best represents the combined income for all family members in your household for the past 12 months before taxes?
    - ☐ Less than $25,000
    - ☐ $25,000 – $49,999
    - ☐ $50,000 – $99,999
    - ☐ $100,000 – $199,999
    - ☐ $200,000 – $299,999
    - ☐ $300,000 or more
    - ☐ I decline to answer
